# The Dietary Furocoumarin Imperatorin Increases Plasma GLP-1 Levels in Type 1-Like Diabetic Rats

**DOI:** 10.3390/nu9111192

**Published:** 2017-10-30

**Authors:** Lin-Yu Wang, Kai-Chun Cheng, Yingxiao Li, Chiang-Shan Niu, Juei-Tang Cheng, Ho-Shan Niu

**Affiliations:** 1 Department of Childhood Education and Nursery, Chia Nan University of Pharmacy and Science, Rende, Tainan City 71710, Taiwan; linyu870203@gmail.com; 2 Division of Pediatrics, Chi-Mei Medical Center, Yong Kang, Tainan City 71003, Taiwan; 3 Department of Medicine, College of Medicine, Kaohsiung Medical University, Kaohsiung City 81201, Taiwan; 4 Department of Psychosomatic Internal Medicine, Kagoshima University Graduate School of Medical and Dental Sciences, Kagoshima 890-8520, Japan; kc-cheng@m3.kufm.kagoshima-u.ac.jp (K.-C.C.); bebeli009@hotmail.com (Y.L.); 5 Department of Medical Research, Chi-Mei Medical Center, Yong Kang, Tainan City 71003, Taiwan; 6 Department of Nursing, Tzu Chi University of Science and Technology, Hualien City 97005, Taiwan; ncs@ems.tcust.edu.tw; 7 Institute of Medical Science, College of Health Science, Chang Jung Christian University, Guei-Ren, Tainan City 71101, Taiwan

**Keywords:** imperatorin, triamterene, GPR119, TGR5, transfection, sitagliptin

## Abstract

Imperatorin, a dietary furocoumarin, is found not only in medicinal plants, but also in popular culinary herbs, such as parsley and fennel. Recently, imperatorin has been shown to activate GPR119 in cells. Another GPR, GPR131, also called TGR5 or G-protein-coupled bile acid receptor 1 (GPBAR1), is known to regulate glucose metabolism. Additionally, TGR5 activation increases glucagon-like peptide (GLP-1) secretion to lower blood sugar levels in animals. Therefore, the present study aims to determine whether the effects of imperatorin on GLP-1 secretion are mediated by TGR5. First, we transfected cultured Chinese hamster ovary cells (CHO-K1 cells) with the TGR5 gene. Glucose uptake was confirmed in the transfected cells using a fluorescent indicator. Moreover, NCI-H716 cells, which secrete GLP-1, were used to investigate the changes in calcium concentrations and GLP-1 levels. In addition, streptozotocin (STZ)-induced type 1-like diabetic rats were used to identify the effects of imperatorin in vivo. Imperatorin dose-dependently increased glucose uptake in CHO-K1 cells expressing TGR5. In STZ diabetic rats, similar to the results in NCI-H716 cells, imperatorin induced a marked increase of GLP-1 secretion that was reduced, but not totally abolished, by a dose of triamterene that inhibited TGR5. Moreover, increases in GLP-1 secretion induced by imperatorin and GPR119 activation were shown in NCI-H716 cells. We demonstrated that imperatorin induced GLP-1 secretion via activating TGR5 and GPR119. Therefore, imperatorin shall be considered as a TGR5 and GPR119 agonist.

## 1. Introduction

Diabetes mellitus (DM) is a metabolic disorder that is characterized by pancreatic islet dysfunction [1]. The prevalence of DM is markedly increased in clinical settings [2]. Generally, type 2 DM (T2DM) is characterized by insulin resistance, as well as hyperglycemia and hyperlipidemia [3]. However, many parameters, including decreased insulin secretion due to pancreatic dysfunction, inadequate hepatic glucose production and peripheral insulin resistance, are involved in the development of T2DM [4]. Therefore, the development of novel therapeutic approaches has been emphasized.

After food ingestion, enteroendocrine cells in the intestinal mucosa release hormones that stimulate insulin secretion from the endocrine pancreas, thus reducing blood glucose levels; this process is known as the incretin effect [5,6]. Two types of incretins, including glucose-dependent insulinotropic polypeptide (GIP) and glucagon-like peptide-1 (GLP-1), have been identified in humans. Physiologically, GLP-1 is primarily produced and released by L-cells located in the distal ileum, whereas GIP is secreted by enteroendocrine K-cells in the proximal gut. Recently, GLP-1 has become a new target for T2DM therapeutics [5]. Two strategies have been widely applied in clinical practice to treat T2DM, namely GLP-1 analogs and inhibitors of the enzyme dipeptidyl peptidase-IV (DPP-4), which degrades both GLP-1 and GIP [5]. However, clinical practice revealed limitations; for example, GLP-1 analogs should be administered by injection only, and the effectiveness of DPP-4 inhibitors is mild [7].

Herbal extracts have been widely applied as complementary and alternative medicines for the treatment of T2DM [8]. Antioxidant-like activity has been considered as the main mechanism(s) of herbal extracts’ effects [9]. Oxidative stress is associated with insulin resistance in T2DM [10]. However, the effective agent(s) of herbal extracts has not been approved for clinical use. Therefore, using herbal extracts to improve insulin resistance or T2DM through the GLP-1 pathway has received increasing attention. Since G-protein-coupled receptor 119 (GPR119) was introduced as a target for treating T2DM and obesity [11], 1500 herbal extracts have been screened for GPR119 activation; imperatorin was evaluated as one of the most promising extracts [12]. Imperatorin, a dietary furocoumarin, is present not only in medicinal plants, but also in popular culinary herbs, such as parsley and fennel. Pharmacological studies have shown that imperatorin has an effect similar to *Angelica dahurica*, or Baizhi in China. The beneficial effects of Baizhi on obesity and fatty liver have been demonstrated in mice [13]. Additionally, imperatorin has also been identified to induce insulin secretion in cells [14]. However, whether imperatorin mediates GLP-1 to cause these effects remains unclear.

GPR131, another GPR, is also known as Takeda G-protein-coupled receptor 5 (TGR5), G-protein-coupled bile acid receptor 1 (GPBAR1) and M-BAR; this GPR has been identified as a bile acid binding receptor [15,16,17]. Activation of TGR5 can increase cyclic adenosine monophosphate (cAMP) levels to activate protein kinase A (PKA) and downstream signaling [15,18]. Interestingly, GLP-1 secretion is induced by TGR5 activation in cultured human intestinal NCI-H716 cells [19]. Therefore, TGR5 agonists have beneficial effects in T2DM-like animals [20]. The benefits of TGR5 activation seem to be similar to those of GPR119 activation [11].

Thus, we are interested in understanding the effects of imperatorin on plasma GLP-1 levels. In the present study, the direct effects of imperatorin on TGR5 are identified by using cultured cells. Additionally, imperatorin-induced increases in plasma GLP-1 levels associated with TGR5 activation are characterized in diabetic rats. Therefore, for the first time, we demonstrate that imperatorin can activate TGR5 and GPR119 sites to promote GLP-1 secretion.

## 2. Materials and Methods

### 2.1. Materials

Imperatorin (purity > 98%) and triamterene (Sigma-Aldrich Chemical Co., St. Louis, MO, USA) were diluted in dimethyl sulfoxide (DMSO) as the stock solution. Additionally, sitagliptin phosphate (Merck, Cramlington, Northumberland, UK), an inhibitor of dipeptidyl peptidase-4 (DPP-4), was diluted in normal saline.

### 2.2. Animals

Male Sprague-Dawley (SD) rats weighing 250–280 g were obtained from the National Laboratory Animal Center (Taipei, Taiwan). Animals used in all experiments were anesthetized with sodium pentobarbital (35 mg/kg, (intra-peritoneally) i.p.) to minimize suffering. The experimental protocols were approved by the Institutional Animal Ethics Committee (103120201) of Chi-Mei Medical Center. All experiments conformed to the Guide for the Care and Use of Laboratory Animals, as well as the guidelines of the Animal Welfare Act. 

### 2.3. Preparation of Diabetic Rats

To induce type 1-like diabetes, streptozotocin (STZ)-diabetic rats were intravenously injected (i.v.) with STZ (65 mg/kg) according to our previous report [21]. Rats were considered to be diabetic if they had a plasma glucose level no less than 300 mg/dL with polyuria and other diabetic features. All studies were started two weeks after the successful induction of diabetes.

### 2.4. Determination of Blood Glucose, Insulin and GLP-1 Levels in Diabetic Rats

Diabetic rats were treated orally with 2 mg/kg/day sitagliptin (a DPP-4 inhibitor) or a vehicle for one week before the administration of the testing substance. Then, following the methods of our previous report [21], we collected blood samples from the femoral vein of anesthetized rats. Similarly, we measured the plasma glucose level using a glucose kit with an automatic analyzer (Quik-Lab, Ames; Miles Inc., Elkhart, IN, USA). Plasma insulin concentrations were estimated using a commercially available enzyme-linked immunosorbent assay (ELISA) kit (Mercodia, Uppsala, Sweden). Additionally, plasma GLP-1 levels were determined using a commercial ELISA kit (EZGLP1T-36K, EMD Millipore Co., Billerica, MA, USA) according to the manufacturer's instructions.

### 2.5. Cell Cultures

We purchased cell lines from the Culture Collection and Research Center of the Food Industry Institute (Hsin-Chiu City, Taiwan). Human NCI-H716 cells (ATCC No. CCL-251) were cultured in RPMI 1640 supplemented with 10% (*v*/*v*) fetal bovine serum (FBS) and 2 mM l-glutamine at 5% CO_2_. Additionally, CHO-K1 cells (ATCC No. CCL-61) were cultured in F-12K growth medium containing 10% (*v*/*v*) FBS. In general, we subcultured the cells once every 3 days using trypsin (GIBCO-BRL Life Technologies, Gaithersburg, MD, USA) and changed the medium every 2–3 days.

### 2.6. TGR5 Transfection into CHO-K1 Cells

Following our previous method [22], we transfected the human TGR5 gene into CHO-K1 cells. The next day, successful transfection was confirmed by Western blots showing bands for TGR5 (32 kDa) and β-actin (43 kDa). Then, the cells expressing TGR5 were treated with imperatorin at the indicated concentrations. 

### 2.7. Measurement of 2-NBDG Uptake in Cells

2-(*N*-(7-nitrobenz-2-oxa-1,3-diazol-4-yl)amino)-2-deoxyglucose (2-NBDG) was used as an indicator of glucose uptake [23]. The experiments were performed as described in a previous report [24] with minor modifications. The fluorescence intensity of the cell suspension was evaluated by using a fluorescence spectrofluorometer (Hitachi F-2000, Tokyo, Japan). Protein concentrations were assayed using a BCA assay kit (Thermo Sci., Rockford, IL, USA). The uptake of 2-NBDG was quantified in cells incubated with imperatorin at the indicated concentrations. For the inhibitor studies, cells were pretreated with triamterene or other inhibitors for 30 min before imperatorin treatment. 

### 2.8. Determination of Intracellular Calcium Concentrations

Changes in intracellular calcium ((Ca^2+^)i) concentrations were detected using the fluorescent probe fura-2 [25]. Fluorescence was recorded continuously by a fluorescence spectrofluorometer (F-2000; Hitachi, Tokyo, Japan). The values of (Ca^2+^)i were then determined as described in a previous report [26]. Background autofluorescence was measured in unloaded cells and was subtracted from all measurements. The values of (Ca^2+^)i were calculated from the fluorescence values measured at 340 nm and 380 nm. Additionally, the effectiveness of the inhibitors, such as triamterene and others, was compared by using a 30-min pretreatment.

### 2.9. GPR119 Silencing in NCI-H716 Cells

We purchased GPR119 small interference RNA (siRNA) (ON-TARGETplus Human GPR119 (139760) siRNA-SMARTpool) from a commercial source (GE Healthcare Dharmacon, Inc., Lafayette, CO, USA). ONT-ARGETplus SMARTpool non-targeting siRNA was used as a negative control. Lipofectamine 2000 (Thermo Fisher Scientific, Pittsburgh, PA, USA) was used to transfer siRNA, according to our previous method [22]. The success of silencing was confirmed by Western blots. After siRNA transfection, NCI-H716 cells were differentiated for another 24 h before assays were performed.

### 2.10. Assay of GLP-1 Secretion from Cells 

NCI-H716 cells (5 × 10^5^ cells per well) were treated with imperatorin at the indicated concentration under 37 °C or 1 h. In some experiments, a 30-min incubation with triamterene was conducted prior to imperatorin treatment. The supernatants were then collected and assayed by using a GLP-1 active ELISA kit (Millipore Co., Billerica, MA, USA). Each assay was performed in duplicate for the indicated samples.

### 2.11. Determination of cAMP Levels in Cells

The cells (5 × 10^5^ cells per well) were treated with imperatorin at the indicated concentrations for 72 h. In some experiments, a 30-min incubation with triamterene was conducted prior to imperatorin treatment. Intracellular cAMP levels were determined using a commercial ELISA kit (Enzo Life Sciences, Farmingdale, NY, USA) according to the manufacturer's instructions. Each assay was performed in duplicate for the indicated samples.

### 2.12. Statistical Analysis

Results are indicated as the mean ± SEM for the sample number (*n*) of each group. One-way analysis of variance (ANOVA) was performed and followed by Tukey’s post hoc test. The datasets of the two sample groups were analyzed by independent *t*-tests. A *p*-value of 0.05 or less was considered significant.

## 3. Results

### 3.1. Imperatorin Activates TGR5 in TGR5-CHO-K1 Cells

**Figure 1:**
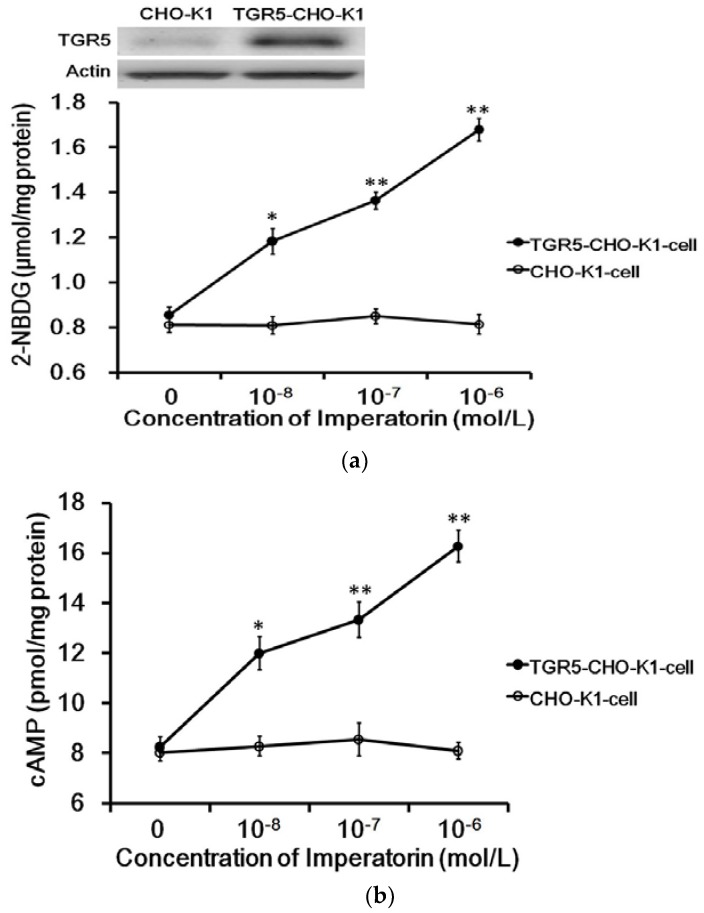
Role of TGR5 activation in imperatorin-induced glucose uptake in cells. (**a**) Successful transfection of CHO-K1 cells with the TGR5 gene was confirmed via Western blotting analysis. Dose-dependent changes in glucose uptake induced by imperatorin in TGR5-transfected CHO-K1 cells (TGR5-CHO-K1 cells) compared with cells transfected with an empty vector (CHO-K1 cells). (**b**) Glucose uptake was measured as described in the Materials and Methods section. Dose-dependent increases in cAMP levels induced by imperatorin in TGR5-transfected CHO-K1 cells (TGR5-CHO-K1 cells) compared with cells transfected with an empty vector (CHO-K1 cells). The cAMP levels were measured with ELISA kits as described in the Materials and Methods section. Values (mean ± SEM) were obtained from eight measurements in each group. * *p* < 0.05 and ** *p* < 0.01 compared with the vehicle-treated group (0 mol/L).

The exogenous TGR5 gene was successfully transfected into CHO-K1 cells and confirmed by Western blots (Figure 1a). The expressed TGR5 receptor in TGR5-CHO-K1 cells was functional, as described in our previous report [22]. Imperatorin markedly increased intracellular 2-NBDG concentrations in cells expressing TGR5. Similarly, as shown in Figure 1b, cyclic AMP (cAMP) levels were also dose-dependently increased by imperatorin in these cells. However, imperatorin failed to produce an effect on 2-NBDG (Figure 1a) and cAMP (Figure 1b) levels in CHO-K1 cells not expressing TGR5. 

### 3.2. Effects of Triamterene-Mediated TGR5 Inhibition on Imperatorin-Induced Glucose Uptake in Cells Expressing TGR5 

**Figure 2:**
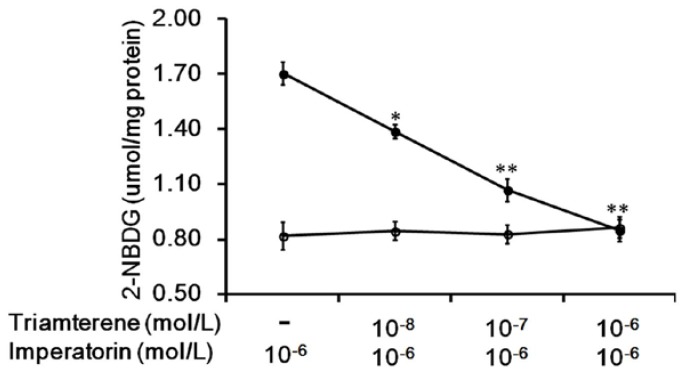
Triamterene inhibits the increased glucose uptake induced by imperatorin in TGR5-transfected cells. The imperatorin-induced increase in glucose uptake (2-NBDG) in TGR5-transfected CHO-K1 cells (TGR5-CHO-K1 cells) was reduced by triamterene in a dose-dependent manner (black circle). However, treatment with triamterene at the same effective dose did not modify spontaneous glucose uptake (open circle). “-” is presented as 0 mol/L. Values (mean ± SEM) were obtained from eight measurements in each group. * *p* < 0.05 and ** *p* < 0.01 compared with imperatorin-stimulated group.

Additionally, the increased 2-NBDG concentrations induced by imperatorin were markedly inhibited in a dose-dependent manner by pretreatment with triamterene (Figure 2). The effects of imperatorin were reversed by triamterene. However, triamterene did not modify the spontaneous uptake of 2-NBDG in these cells expressing TGR5 (Figure 2).

### 3.3. Effects of Imperatorin on GLP-1 Secretion in Cultured Cells

**Figure 3:**
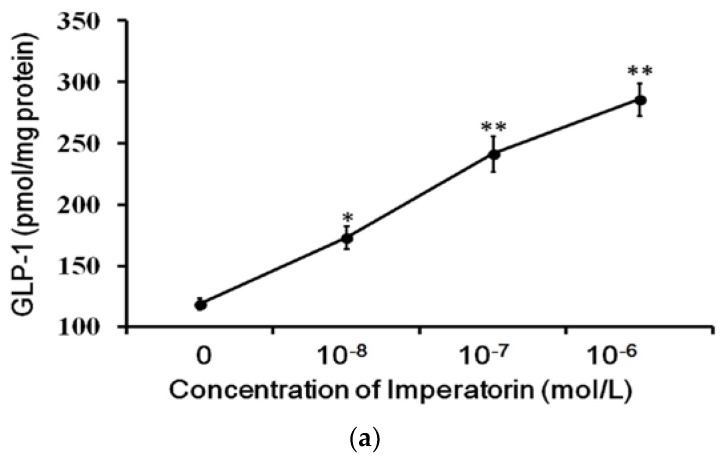

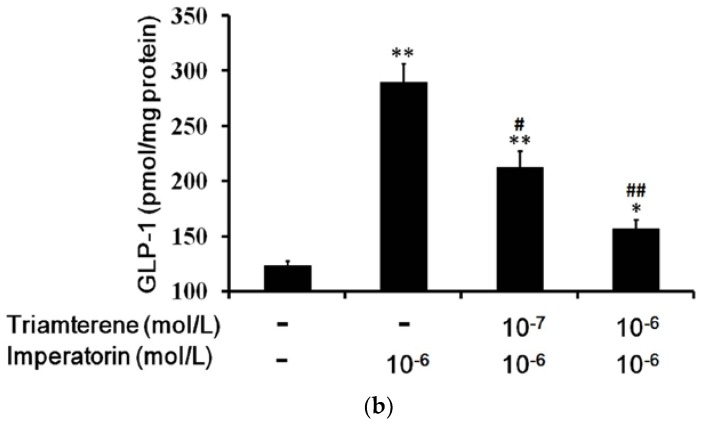
Increased GLP-1 secretion induced by imperatorin in NCI-H716 cells. (**a**) Imperatorin markedly induced GLP-1 secretion. (**b**) Triamterene dose-dependently inhibited imperatorin-induced GLP-1 secretion in NCI-H716 cells. Values (mean ± SEM) were obtained from eight measurements in each group. * *p* < 0.05 and ** *p* < 0.01 compared with the vehicle-treated group (basal value). “-” is presented as 0 mol/L. ^#^ *p* < 0.05 and ^##^ *p* < 0.01 compared with the vehicle-treated, imperatorin-stimulated group (second column).

The cultured intestinal NCI-H716 cell line that expresses TGR5 receptors is widely used to study GLP-1 secretion [27]. Therefore, we used NCI-H716 cells to identify the effects of imperatorin on GLP-1 secretion. A dose-dependent increase in GLP-1 secretion induced by imperatorin was also observed in NCI-H716 cells (Figure 3). This result is consistent with the previously described stimulation of GLP-1 secretion through TGR5 activation by another agonist [22,28,29]. The effects of imperatorin were inhibited in a dose-dependent manner by pretreatment with triamterene. However, triamterene did not completely abolish the effects of imperatorin in NCI-H716 cells.

### 3.4. Effect of Imperatorin on Plasma Glucose Levels in Diabetic Rats

**Figure 4:**
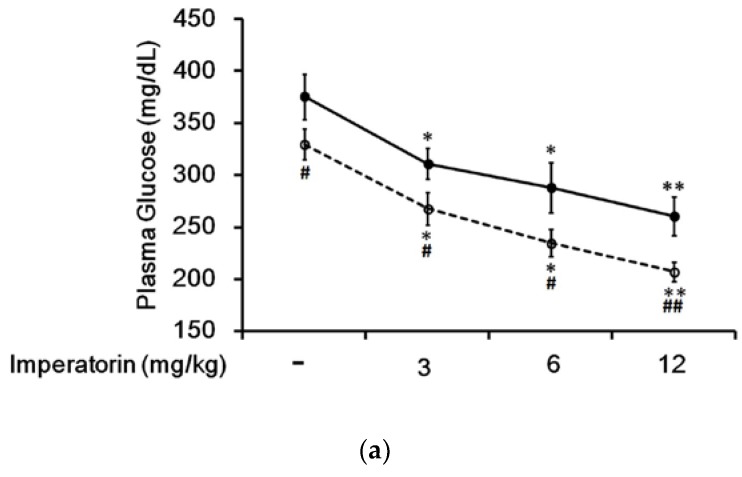

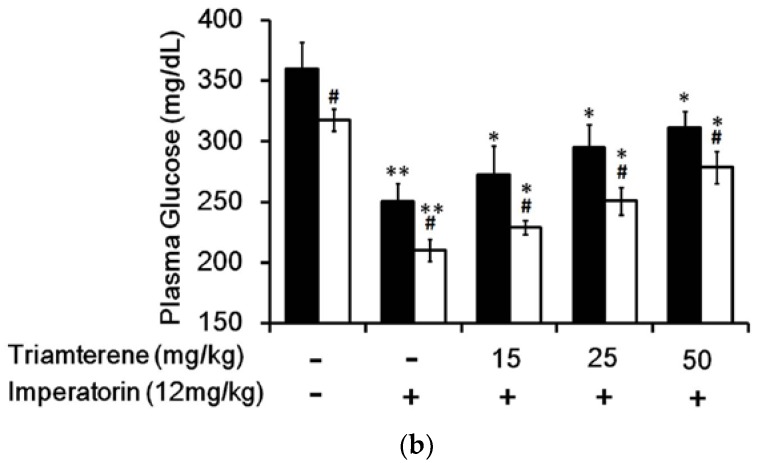
Effects of imperatorin on plasma glucose levels in type 1-like diabetic rats. (**a**) Plasma glucose levels were determined 1 h after the administration of imperatorin at the indicated dose in the vehicle-treated group (solid line) or the sitagliptin-treated (5 mg/kg/day orally for 14 days) group (broken line). (**b**) The inhibitory effects of triamterene on imperatorin-induced reductions in plasma glucose levels in diabetic rats receiving vehicle (black column) or sitagliptin (open column) at 5 mg/kg/day orally for 14 days. These diabetic rats were pretreated with triamterene at the indicated dose for 30 min. The values are expressed as the mean ± SEM obtained from eight samples per group. “-” is presented as 0 mol/L; “+” is presented as imperatorin treatment. * *p* < 0.05 and ** *p* < 0.01 compared with the vehicle-treated group. ^#^ *p* < 0.05 and ^##^ *p* < 0.01 compared with the basal group without sitagliptin treatment.

In STZ-induced diabetic rats, an established type 1-like diabetic model, imperatorin dose-dependently attenuated hyperglycemia (Figure 4a). Additionally, hyperglycemia was reduced in these diabetic rats by sitagliptin treatment at an effective dose to inhibit dipeptidyl peptidase-4 (DPP-4). As shown in Figure 4a, the decrease in hyperglycemia induced by imperatorin was also markedly enhanced by sitagliptin treatment in these diabetic rats. Moreover, triamterene dose-dependently inhibited the effects of imperatorin in diabetic rats. As shown in Figure 4b, hyperglycemia attenuated by imperatorin was dose-dependently inhibited by triamterene in diabetic rats that received sitagliptin. However, triamterene did not completely reverse the effects of imperatorin in diabetic rats that received sitagliptin or not.

### 3.5. Effects of Imperatorin on Plasma GLP-1 Levels in Diabetic Rats

**Figure 5:**
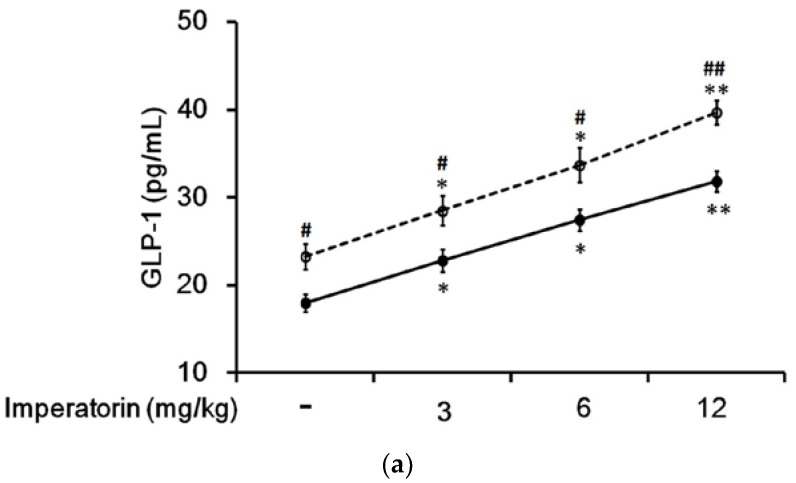

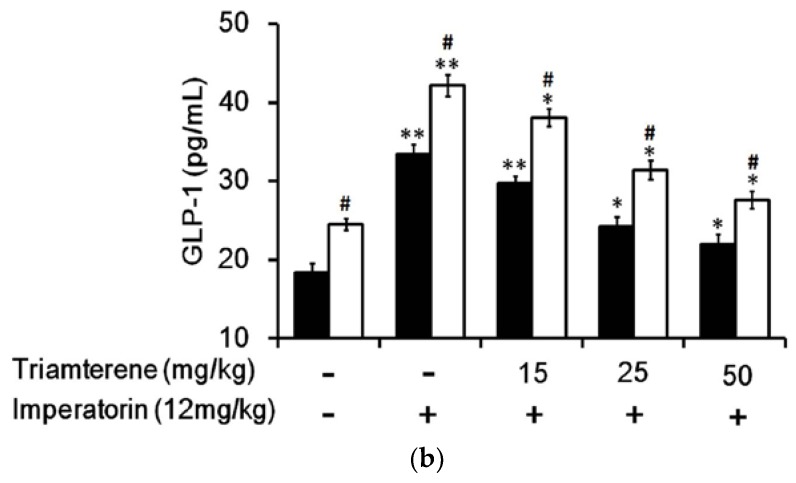
Effects of imperatorin on plasma GLP-1 levels in type 1-like diabetic rats. (**a**) Plasma GLP-1 levels were determined 45 min after the administration of imperatorin at the indicated dose in the vehicle-treated group (solid line) or the sitagliptin-treated (5 mg/kg/day orally for 14 days) group (broken line). (**b**) Triamterene inhibited the imperatorin-induced increases in plasma GLP-1 levels in diabetic rats that received vehicle (black column) or 5 mg/kg/day sitagliptin (open column) orally for 14 days. The diabetic rats were pretreated with triamterene at the indicated dose for 30 min. The values are expressed as the mean ± SEM obtained from eight samples per group. “-” is presented as 0 mol/L; “+” is presented as imperatorin treatment. * *p* < 0.05 and ** *p* < 0.01 compared with the vehicle-treated group. ^#^ *p* < 0.05 and ^##^ *p* < 0.01 compared with the basal group without sitagliptin treatment.

In STZ-induced diabetic rats, imperatorin dose-dependently increased plasma GLP-1 levels (Figure 5a). The increase in plasma GLP-1 levels should be more pronounced in diabetic rats treated with sitagliptin at a dose sufficient to inhibit DPP-4, the GLP-1 inactivating enzyme. Accordingly, as shown in Figure 5a, the imperatorin-induced increases in plasma GLP-1 levels were markedly enhanced by sitagliptin treatment in diabetic rats. Moreover, as shown in Figure 5b, increases in plasma GLP-1 levels induced by imperatorin were also dose-dependently inhibited by triamterene in diabetic rats treated with or without sitagliptin. However, similar to the hyperglycemia changes, triamterene did not completely reverse the imperatorin-induced GLP-1 increases in diabetic rats.

### 3.6. Effects of Imperatorin on Plasma Insulin Levels in Diabetic Rats

**Figure 6:**
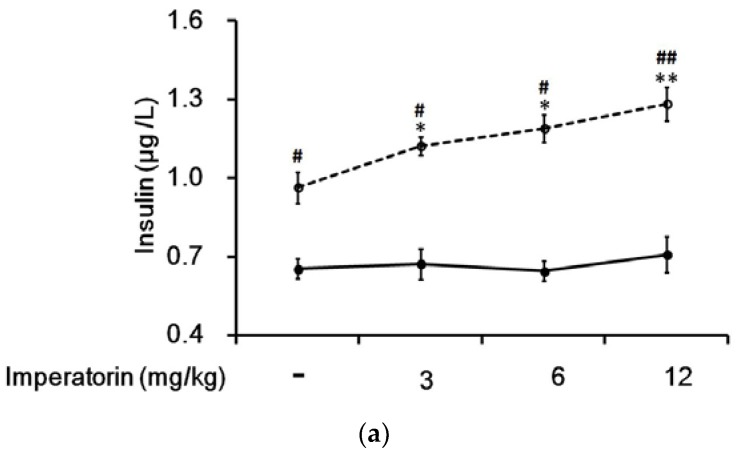

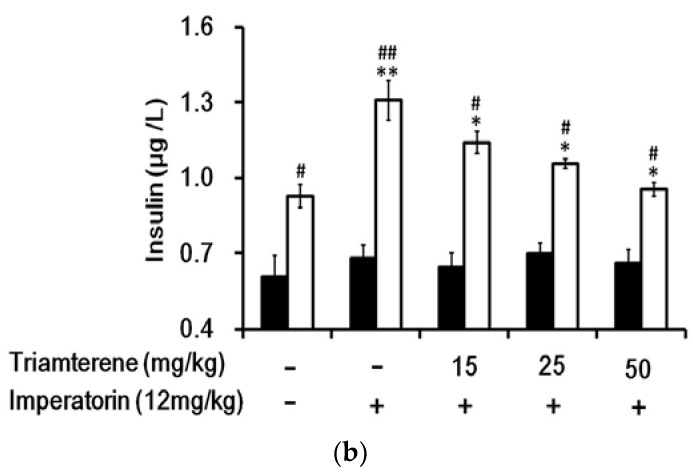
Effects of imperatorin on the plasma insulin levels in type 1-like diabetic rats. (**a**) The plasma insulin levels were determined 50 min after the administration of imperatorin at the indicated dose in the vehicle-treated group (solid line) or in the sitagliptin-treated (5 mg/kg/day orally for 14 days) group (broken line). (**b**) Triamterene inhibited the imperatorin-induced increase in the plasma insulin levels in diabetic rats that received vehicle (black column) or 5 mg/kg/day sitagliptin (open column) orally for 14 days. The diabetic rats were pretreated with triamterene at the indicated dose for 30 min. The values are expressed as the mean ± SEM obtained from eight samples per group. “-” is presented as 0 mol/L. “+” is presented as imperatorin treatment.* *p* < 0.05 and ** *p* < 0.01 compared with the vehicle-treated group. ^#^ *p* < 0.05 and ^##^ *p* < 0.01 compared with the basal group without sitagliptin treatment.

In STZ-induced diabetic rats, the residual insulin levels in plasma were not affected by imperatorin (Figure 6a). Interestingly, the plasma insulin levels were markedly increased in the diabetic rats that received two weeks of treatment with sitagliptin at a dose sufficient to inhibit DPP-4, the GLP-1 inactivating enzyme. In addition, the insulin levels were further increased in a dose-dependent manner by imperatorin (Figure 6a). Moreover, as shown in Figure 6b, the increase in plasma insulin levels induced by imperatorin was also dose-dependently inhibited by triamterene in the diabetic rats that received sitagliptin. However, similar to the results above, triamterene did not totally reverse the increased insulin levels induced by imperatorin in the diabetic rats.

### 3.7. Role of GPR119 in Increased GLP-1 Secretion Induced by Imperatorin in Cells

**Figure 7:**
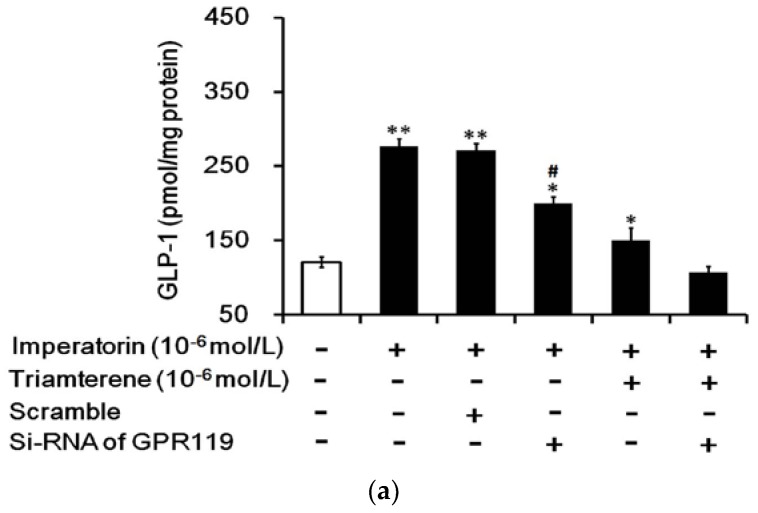

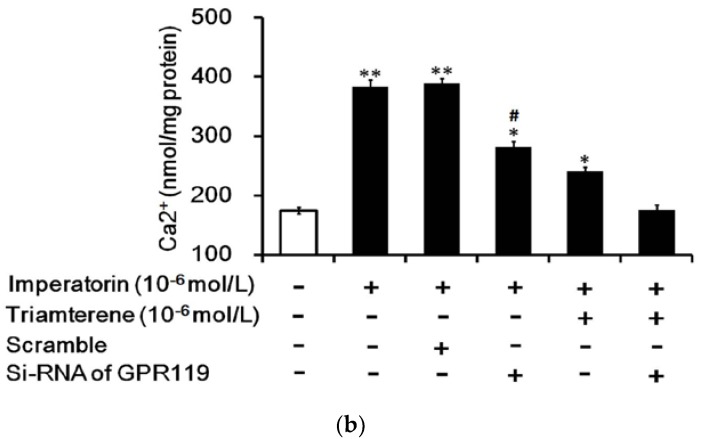
GPR119 is involved in increased GLP-1 secretion induced by imperatorin in NCI-H716 cells. The successful transfection of cells with GPR119 siRNA was confirmed using Western blots. (**a**) The inhibition of imperatorin-induced GLP-1 secretion by triamterene was completely abolished when GPR119 was knocked down with siRNA in NCI-H716 cells. (**b**) The increase in intracellular calcium ((Ca^2+^)i) levels was also blunted in NCI-H716 cells. The values (mean ± SEM) were obtained from eight measurements in each group. “-” in all groups are presented as 0 mol/L. “+” is present as the administration of drugs at the indicated dose. * *p* < 0.05 and ** *p* < 0.01 compared with the vehicle-treated group (first column). ^#^ *p* < 0.05 compared with the scrambled siRNA-treated and imperatorin-stimulated group (third column).

As stated above, triamterene did not completely abolish the effects of imperatorin in NCI-H716 cells and STZ-diabetic rats. It has been demonstrated that imperatorin can activate GPR119 [12]. Therefore, we examined GPR119 as another factor in imperatorin-induced GLP-1 secretion. Using siRNA specific to GPR119, we silenced GPR119 expression in NCI-H716 cells to evaluate changes in the effects of imperatorin. The residual effect of imperatorin on GLP-1 secretion was fully abolished in the absence of GPR119 after treatment with triamterene at a dose sufficient to block TGR5 (Figure 7a). Similar changes in the effects of imperatorin on calcium levels were also observed (Figure 7b). Therefore, the effects of imperatorin seem to be associated with the activation of both TGR5 and GPR119.

## 4. Discussion

In the present study, we found that imperatorin can activate TGR5 in both cells and animals. We demonstrated the direct effect of imperatorin on TGR5 using CHO-K1-cells transiently transfected with the TGR5 gene. Additionally, the effects of imperatorin on TGR5 were observed in cultured cells that secrete GLP-1. Then, we confirmed the increase in plasma GLP-1 levels induced by imperatorin via TGR5 activation by showing that GLP-1 levels were markedly decreased by the TGR5 inhibitor triamterene in diabetic rats. Therefore, imperatorin should be considered as a TGR5 agonist both in vitro and in vivo.

Until now, in addition to its role in metabolic regulation, TGR5 has been shown to be involved in many functions of biological systems [30]. Therefore, many studies have focused on the development of TGR5 agonists [31]. However, the TGR5 agonists are not used in clinical applications. In this report, we demonstrated for the first time that the stimulatory effects of imperatorin on TGR5 increase GLP-1 secretion. 

The direct effects of imperatorin on TGR5 were first identified in CHO-K1-cells transiently transfected with the TGR5 gene. Imperatorin increased glucose uptake in cells expressing TGR5. Additionally, imperatorin increased the levels of cAMP, which serves as a TGR5 signal in these cells. Neither of these effects of imperatorin can be observed in CHO-K1 cells not expressing TGR5. Thus, imperatorin has been shown to mediate TGR5. These results are consistent with those of our previous report [22] that examined another TGR5 agonist. Moreover, triamterene also attenuated the effect of imperatorin in a dose-dependent manner. In a recent study, triamterene specifically blocked TGR5 [32]. Therefore, imperatorin can activate TGR5 in vitro.

The effects of imperatorin were further investigated in NCI-H716 cells, which are widely used for the study of GLP-1 secretion [22]. TGR5 activation can induce GLP-1 secretion via cyclic AMP to enhance calcium influx in this cell line [22]. In the present study, we confirmed that imperatorin increases GLP-1 secretion from NCI-H716 cells. This result is consistent with the effect of another TGR5 agonist, betulinic acid, shown in a previous report [22]. Moreover, this effect of imperatorin was inhibited by triamterene. It has been reported that triamterene possesses a TGR5 blocking-like action as a pleiotropic effect in addition to K^+^-sparing [32]. The functional blockade of TGR5 by triamterene supports that imperatorin mediates TGR5. Therefore, imperatorin has been identified as a TGR5 agonist, similar to other compounds [33].

GLP-1 regulates plasma glucose levels through several mechanisms, including insulin secretion stimulation and proinsulin gene expression, β-cell proliferation and anti-apoptosis increases [34]. Therefore, we investigated the in vivo effects of imperatorin using STZ-induced diabetic rats. In this animal model, the mediation of insulin is negligible as described previously [21]. Imperatorin dose-dependently attenuated hyperglycemia in diabetic rats. This effect of imperatorin was enhanced by sitagliptin at a dose effective for inhibiting DPP-4, the GLP-1 inactivating enzyme [35]. Thus, it should be considered that the antihyperglycemic effects of imperatorin are associated with GLP-1. It has been established that GLP-1 increases insulin secretion during meal ingestion, thereby reducing postprandial glucose levels [36]. However, the physiological role of GLP-1 has recently been expanded beyond its well-recognized effects on pancreatic β-cells [37]. In addition to reports that GLP-1 directly stimulates hepatic glucose uptake [38] and suppresses hepatic glucose production [39], several gastrointestinal effects of GLP-1 have been shown to lower glucose levels without insulin [40]. Therefore, we observed a reduction in hyperglycemia induced by imperatorin in STZ-induced diabetic rats. Moreover, after DPP-4 inhibition for two weeks, plasma insulin levels were markedly increased in these diabetic rats. These results are consistent with the view that GLP-1 levels increased by DPP-4 inhibition may have beneficial effects on pancreatic islets [41]. Plasma insulin levels were further increased by imperatorin in diabetic rats. Additionally, triamterene dose-dependently inhibited this effect of imperatorin indicating the mediation of TGR5 activation. Assays of plasma GLP-1 levels also showed a dose-dependent increase caused by imperatorin in type 1-like diabetic rats. The inhibition of DPP-4 by sitagliptin resulted in a marked increase in plasma GLP-1 levels induced by imperatorin in diabetic rats. Sitagliptin is a clinical antidiabetic drug that inhibits the degradation of GLP-1 [35]. The effects of imperatorin on plasma GLP-1, insulin and glucose levels when DPP-4 was blocked were dose-dependently inhibited by triamterene. These results are consistent with a report in diabetic patients [42] showing that the activation of GPR131 (TGR5) induces GLP-1 secretion. Therefore, we suggest that imperatorin can activate TGR5, which may increase plasma GLP-1 levels to lower hyperglycemia in diabetic rats. However, the effects of imperatorin were not totally abolished by triamterene, indicating the involvement of another mechanism. GPR119 activation by imperatorin [12] seems most likely, as described previously [11]. Because a GPR119-specific inhibitor is not available, we thus used siRNA to silence the expression of GPR119 in cells. In agreement with our hypothesis, the effects of imperatorin were completely abolished in cells transfected with GPR119 siRNA after treatment with triamterene at a dose sufficient to block TGR5. Therefore, we identified that the effects of imperatorin were caused by the activation of both TGR5 and GPR119. However, this hypothesis needs to be confirmed in GPR119-knockdown animals in the future. 

## 5. Conclusions

We found that imperatorin can activate TGR5 in animals and demonstrated for the first time that imperatorin increases GLP-1 secretion through TGR5 and GPR119 activation in cells. Therefore, imperatorin should be considered as an agonist of both TGR5 and GPR119 for progression from bench to bedside in the future.
